# APC^cdh1^ Mediates Degradation of the Oncogenic Rho-GEF Ect2 after Mitosis

**DOI:** 10.1371/journal.pone.0023676

**Published:** 2011-08-19

**Authors:** Caroline Liot, Laetitia Seguin, Aurélie Siret, Catherine Crouin, Susanne Schmidt, Jacques Bertoglio

**Affiliations:** 1 Inserm U749, Institut Gustave Roussy, Villejuif, France; 2 CNRS UMR 5237, Montpellier, France; University Paris Sud, France

## Abstract

**Background:**

Besides regulation of actin cytoskeleton-dependent functions, Rho GTPase pathways are essential to cell cycle progression and cell division. Rho, Rac and Cdc42 regulate G1 to S phase progression and are involved in cytokinesis. RhoA GDP/GTP cycling is required for normal cytokinesis and recent reports have shown that the exchange factor Ect2 and the GTPase activating protein MgcRacGAP regulate RhoA activity during mitosis. We previously showed that the transcription factors E2F1 and CUX1 regulate expression of MgcRacGAP and Ect2 as cells enter S-phase.

**Methodology/Principal Findings:**

We now report that Ect2 is subject to proteasomal degradation after mitosis, following ubiquitination by the APC/C complex and its co-activator Cdh1. A proper nuclear localization of Ect2 is necessary for its degradation. APC-Cdh1 assembles K11-linked poly-ubiquitin chains on Ect2, depending upon a stretch of ∼25 amino acid residues that contain a bi-partite NLS, a conventional D-box and two TEK-like boxes. Site-directed mutagenesis of target sequences generated stabilized Ect2 proteins. Furthermore, such degradation-resistant mutants of Ect2 were found to activate RhoA and subsequent signalling pathways and are able to transform NIH3T3 cells.

**Conclusions/Significance:**

Our results identify Ect2 as a bona fide cell cycle-regulated protein and suggest that its ubiquitination-dependent degradation may play an important role in RhoA regulation at the time of mitosis. Our findings raise the possibility that the overexpression of Ect2 that has been reported in some human tumors might result not only from deregulated transcription, but also from impaired degradation.

## Introduction

Rho GTPase pathways control the actin cytoskeleton and are thus involved in a wide array of cellular functions including cell morphology, cell migration and regulation of gene expression. They are also essential to cell cycle progression and cell division [1], [2]. Rho, Rac and Cdc42 regulate G1 to S phase progression and are involved in cytokinesis. Failed cytokinesis can occur either as a result of Rho inhibition or from expressing constitutively activated versions of the GTPase, indicating that GDP/GTP cycling is required for normal cytokinesis [3], [4], [5]. Consistent with this hypothesis, recent reports have shed light on the complementary roles of at least two exchange factors, GEF-H1 and Ect2, which control the loading of GTP onto Rho and of two GTPase activating proteins (GAPs), p190RhoGAP and MgcRacGAP, which increase the rate of GTP hydrolysis [6], [7], [8], [9]. Although the precise timing of activation and interplay between these Rho regulatory proteins remain to be deciphered, they all appeared to be critical for mitosis and cytokinesis. It is of interest that these proteins are phosphorylated by the mitotic kinases CDK1, Polo Kinase and Aurora B that appear to regulate their conformation and/or catalytic activity [6], [10], [11], [12], [13].

In many cell types, SiRNA silencing of either Ect2 or MgcRacGAP results in multinucleated cells. During mitosis, MgcRacGAP localizes to the central spindle whereas Ect2 is found both at the spindle and at the cortical plasma membrane. They act to regulate the activity of RhoA which controls contraction of the actomyosin ring and ingression of the cleavage furrow. Genes whose protein products play specific roles in cell cycling and exert their functions at defined steps of cycle progression are often regulated in a cell cycle-dependent manner. We previously showed that expression of MgcRacGAP and Ect2 is upregulated when cells enter S-phase and found that the transcription factors E2F1 and CUX1 are responsible for transcriptional induction of these genes [14], [15].

Mitosis progression is an intricate process which critically depends upon activity of the ubiquitin ligase known as the APC/C (Anaphase promoting complex/Cyclosome). Indeed APC/C mediates ubiquitination, thus triggering subsequent proteasome-dependent degradation of key proteins, such as securin or cyclins A and B, allowing progression from prometaphase to mitotic exit [16]. The APC is fully active as a ubiquitin ligase once it has bound to its co-activators Cdc20 or Cdh1, resulting in distinct assemblies named APC^Cdc20^ or APC^Cdh1^. APC^Cdc20^ activity is critical for metaphase/anaphase transition, whereas APC^Cdh1^ is fully active in late mitosis and G1 phase [16].

We now report that Ect2 is degraded after mitosis, depending upon the ubiquitin ligase activity of the APC/C complex and its co-activator Cdh1. We identified a stretch of 25 residues towards the c-terminus end of Ect2 whose mutations generated stabilized Ect2 proteins. These mutated Ect2 proteins that are less prone to degradation were found to be able to transform NIH3T3 cells. We speculate that the reported over expression of Ect2 in some human tumors might result not only from deregulated transcription, but also from impaired degradation.

## Results

### Ect2 levels fluctuate during the cell cycle

**Figure 1:**
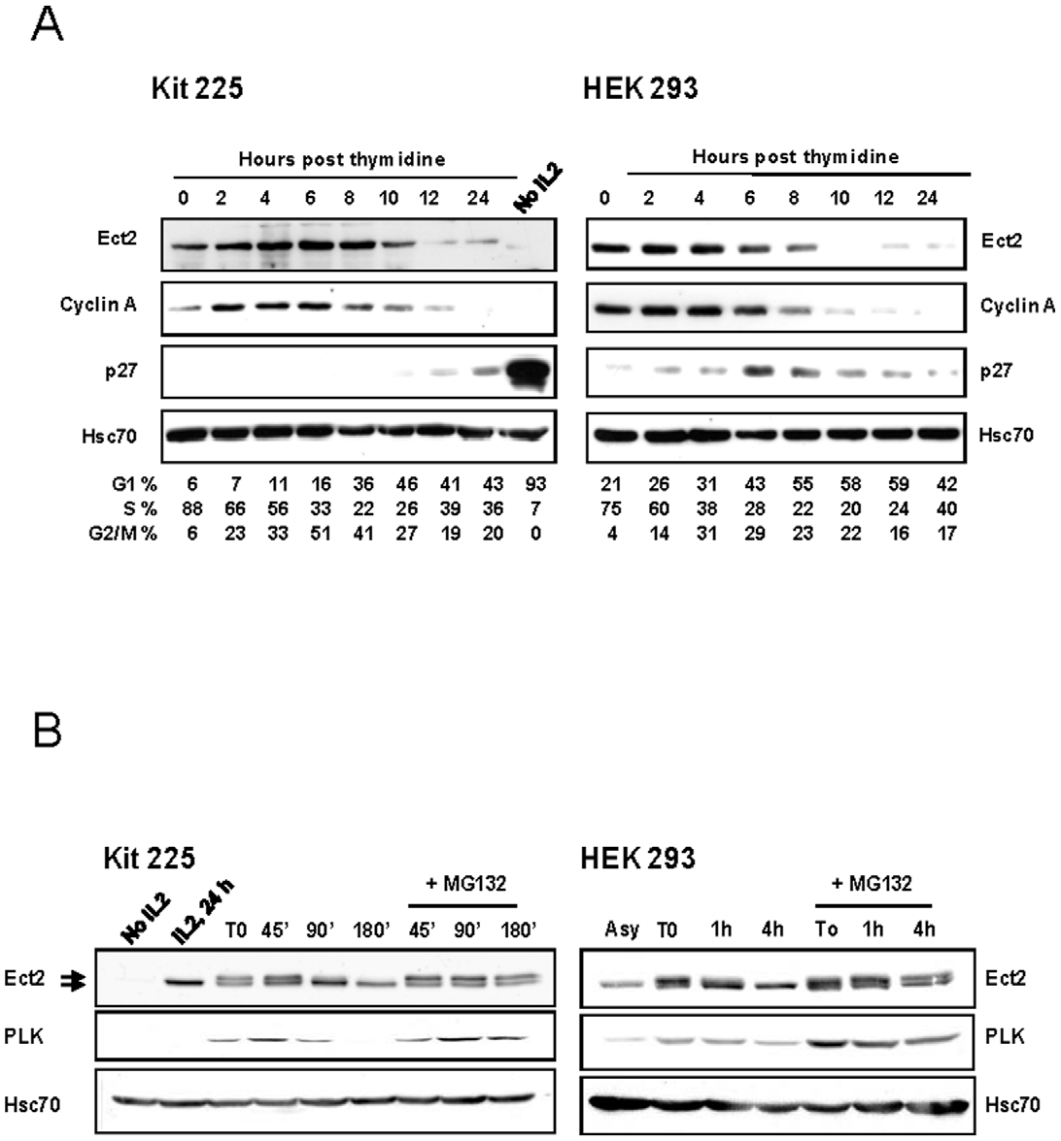
Expression levels of Ect2 fluctuate during the cell cycle. (**A**) IL2-dependent Kit 225 cells and HEK 293 cells were synchronized in S phase by a double thymidine block and then released in fresh medium. Variations in expression of Ect2, compared to the G1/S markers p27 and cyclin A were analyzed by western blot at the indicated times. Hsc70 is shown as a loading control. Numbers shown underneath the figure represent the % of cells in G1, S or G2/M as assessed by FACS analysis of cells stained with propidium iodide. (**B**) *Degradation of Ect2 at the end of mitosis depends on the proteasome*: Cells were blocked in prometaphase in the presence of nocodazole (noted as time 0, T0) then released by washing in medium in the absence or presence of MG132 (20 µM). Cell lysates were analyzed by western blot with the indicated antibodies.

To study expression of Ect2 during the cell cycle, western blot analyses were performed in two different cell types: the interleukin-2 (IL2)-dependent human lymphocyte cell line Kit 225 and the HEK 293 epithelial adherent cell line. Both cell lines were synchronized in S-phase by a double thymidine block. Ect2 is undetectable in Kit 225 cells arrested in G1 by IL2 deprivation (Figure 1A, left panel, right hand side, No IL2). Using the double thymidine block procedure, Kit 225 cells appear to arrest in early S-phase. Following release in fresh medium, Ect2 expression increases to reach a maximum at 4 to 6 hours when cells peak in G2/M. As cells exit from mitosis Ect2 decreases, in parallel to Cyclin A expression. A similar variation in Ect2 expression was observed in HEK 293 cells which however appear to arrest later in S-phase than Kit 225 cells as suggested by the level of cyclin A, and peak earlier in mitosis (4–6 hours as compared to 6–8 hours for Kit 225) (Figure 1A).

To analyze the behaviour of Ect2 during mitosis, extracts were prepared from cells arrested in prometaphase by a nocodazole treatment or at various times following release from the nocodazole block. Ect2 is known to be regulated by serine/threonine phosphorylation during mitosis [12], [17], and appears as a doublet in nocodazole-arrested cells as compared to a single band in cells that were harvested during S-phase (after 24 hours in culture with IL2). Under these experimental conditions, both Kit 225 and HEK 293 exit mitosis, as assessed by propidium iodide staining (not shown), and enter G1 within 90 minutes of release from the nocodazole block. At this time, a progressive decrease in intensity of the shifted (phosphorylated) band of Ect2 was observed, suggesting that the protein either became dephosphorylated, or degraded (Figure 1B). Given that we had observed that Ect2 disappeared at the end of the cell cycle (Figure 1A), the second hypothesis appeared more likely, and it was directly assessed by incubating cells in presence of MG132, a 26S proteasome inhibitor. Indeed, in the presence of MG132, expression of Ect2 was maintained, indicating that proteasome-dependent degradation might be responsible for its disappearance. These observations were made in both Kit 225 and HEK 293 cells, and expression of Polo kinase was used as a control for mitosis progression.

### Ect2 is a substrate for APC^Cdh1^

**Figure 2:**
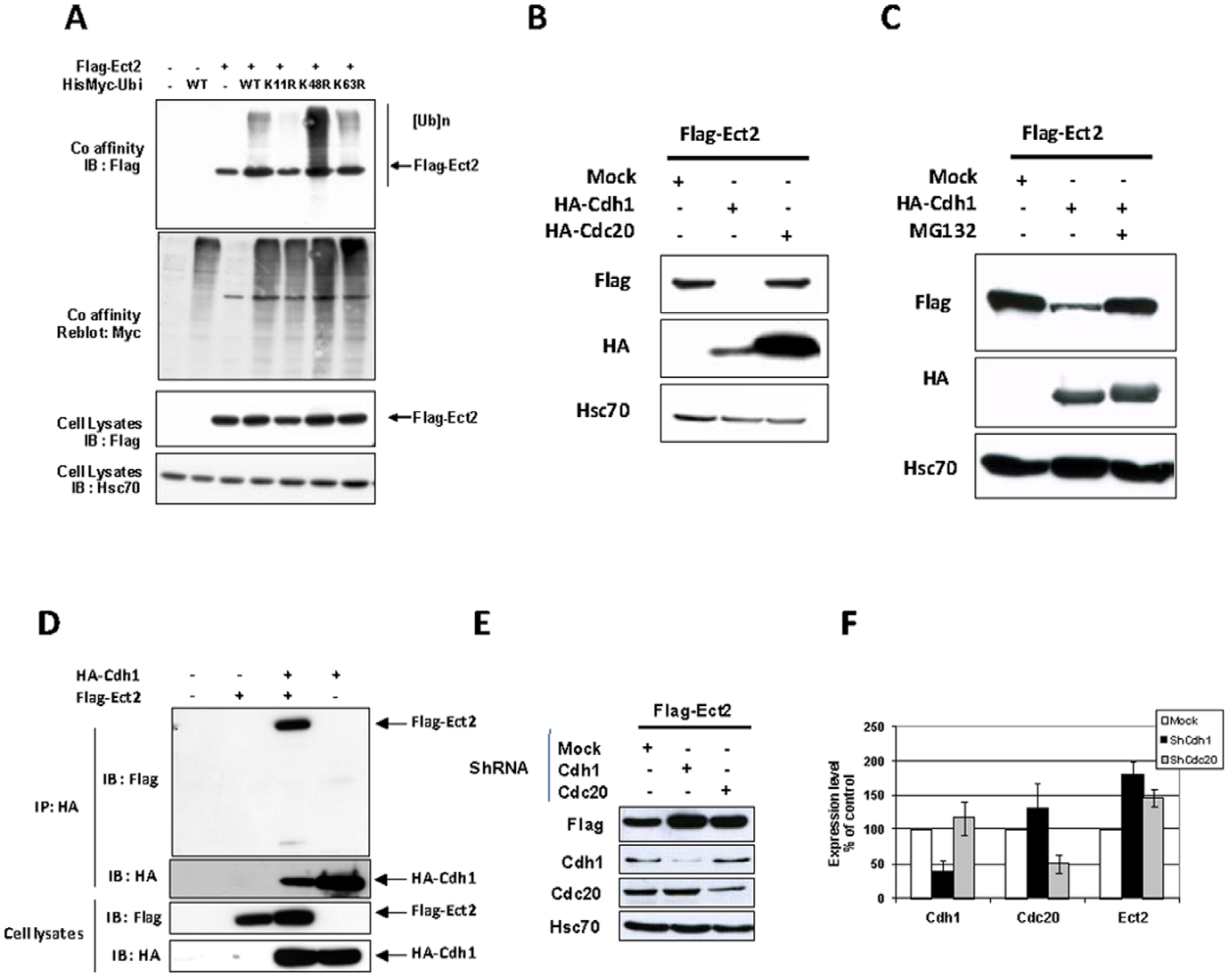
Degradation of Ect2 is mediated by APC/Cdh1. (**A**) HEK 293 cells were co-transfected with expression vectors for Flag-Ect2 and His-tagged ubiquitin either wild type or mutated on lysine residues 11, 48 or 63. Cells were cultured for 24 hrs, with MG132 being added for the last 6 h. Ubiquitinated proteins were enriched on a chelating column loaded with cobalt (Co) and analyzed by western blotting with anti-Flag. The membrane was then reblotted with anti-Myc antibodies. (**B**) *Overexpression of Cdh1, but not Cdc20, increases degradation of Ect2*: HEK 293 cells were co-transfected with expression vectors for Flag-Ect2 and either HA-tagged Cdh1 or Cdc20. After 24 hrs in culture, cell lysates were prepared and analyzed by western blots with anti-Flag and control antibodies. (**C**) *MG132 prevents Cdh1-mediated degradation of Ect2*: Cells were treated as in (B), except that MG132 (20 µM) was added for the last 16 hrs of culture. (**D**) *Ect2 interacts with Cdh1*: HEK 293 cells were co-transfected with expression vectors for Flag-Ect2 and HA-tagged Cdh1. Cells were cultured for 24 hrs, with MG132 being added for the last 16 h. Cell lysates were then prepared and immunoprecipitated with anti HA antibodies. Proteins recovered on protein G-agarose beads were analyzed by western blots with anti-Flag antibodies. (**E**) *ShRNA knockdown of Cdh1 stabilizes Ect2*: HEK 293 cells were co-transfected with expression vectors for Flag-Ect2 and either pSuper Cdh1 or pSuper Cdc20. Cell lysates were analyzed by western blot with anti-Flag and control antibodies. (**F**) Densitometry scanning quantification of 3 independent experiments as the one shown in (E).

Proteasome-dependent degradation depends upon ubiquitination of the substrate. Target proteins are first modified by the covalent attachment of a ubiquitin molecule on a lysine residue. Additional molecules of ubiquitin can subsequently be attached to one of the seven lysines of the previously cross-linked ubiquitin molecule, leading to the formation of polyubiquitin chains. By co-expressing tagged Ect2 together with a HisMyc-ubiquitin expression vector we demonstrated that Ect2 could indeed be ubiquitinated in HEK293 cells (Figure 2A). Furthermore, the use of HisMyc-ubiquitin mutated on lysine 11, but not on lysine 48 or 63, prevented detection of poly-ubiquitinated Ect2, whereas reblotting with anti-Myc showed that the total amount of ubiquitinated proteins that can be recovered on cobalt-loaded beads was similar, regardless of the specific mutations in ubiquitin.

Ubiquitin ligases direct the assembly of poly-ubiquitin chains of diverse topologies, with distinct consequences on the fate of the substrate, and it has been shown that APC/C preferentially assembles K11-linked chains [18], [19]. The results shown in Figure 2A indicated that poly-ubiquitination of Ect2 is critically dependent upon lysine 11 in ubiquitin. Together with the timing of Ect2 degradation these results suggested that APC/C may be the ubiquitin ligase targeting Ect2. To directly investigate this point, we aimed at interfering with expression of Cdh1 or Cdc20. In a first series of experiments, HEK 293 cells were co-tranfected with expression plasmids for HA-tagged Cdh1 or Cdc20 together with Flag-Ect2. Overexpression of Cdh1, but not of Cdc20, resulted in a dramatic decrease in expression of Ect2 (Figure 2B). Thus, these results suggest that Cdh1 may be the active co-activator of APC in this process. Of note, addition of the proteasome inhibitor MG132 during the experiment rescued Ect2 expression from the effects of Cdh1 overexpression, demonstrating the specificity of the observed effects (Figure 2C). Although we could not define experimental conditions allowing the demonstration that endogenous Ect2 and Cdh1 interact with each other, we were able to show such an interaction by co-immunoprecipitation in HEK 293 cells transfected to overexpress Flag-Ect2 and HA-Cdh1 (Figure 2D). Finally, we used small hairpin ShRNA targeting either Cdh1 or Cdc20 to reduce their expression [20]. Under our experimental conditions, around 50% reduction in the amount of Cdh1 or Cdc20 proteins detectable by western blot was reproducibly achieved. Consistent with the observations above, knockdown of Cdh1 resulted in accumulation of Flag-Ect2 (Figure 2E and 2F). Not unexpectedly, Cdc20 knockdown, that should impair full activation of Cdh1, also resulted in some increase in the level of Ect2. Altogether, the series of experiments shown in Figure 2 clearly demonstrated that Ect2 is targeted for degradation by APC^Cdh1^.

### A proper nuclear localization is required for Ect2 degradation

**Figure 3:**
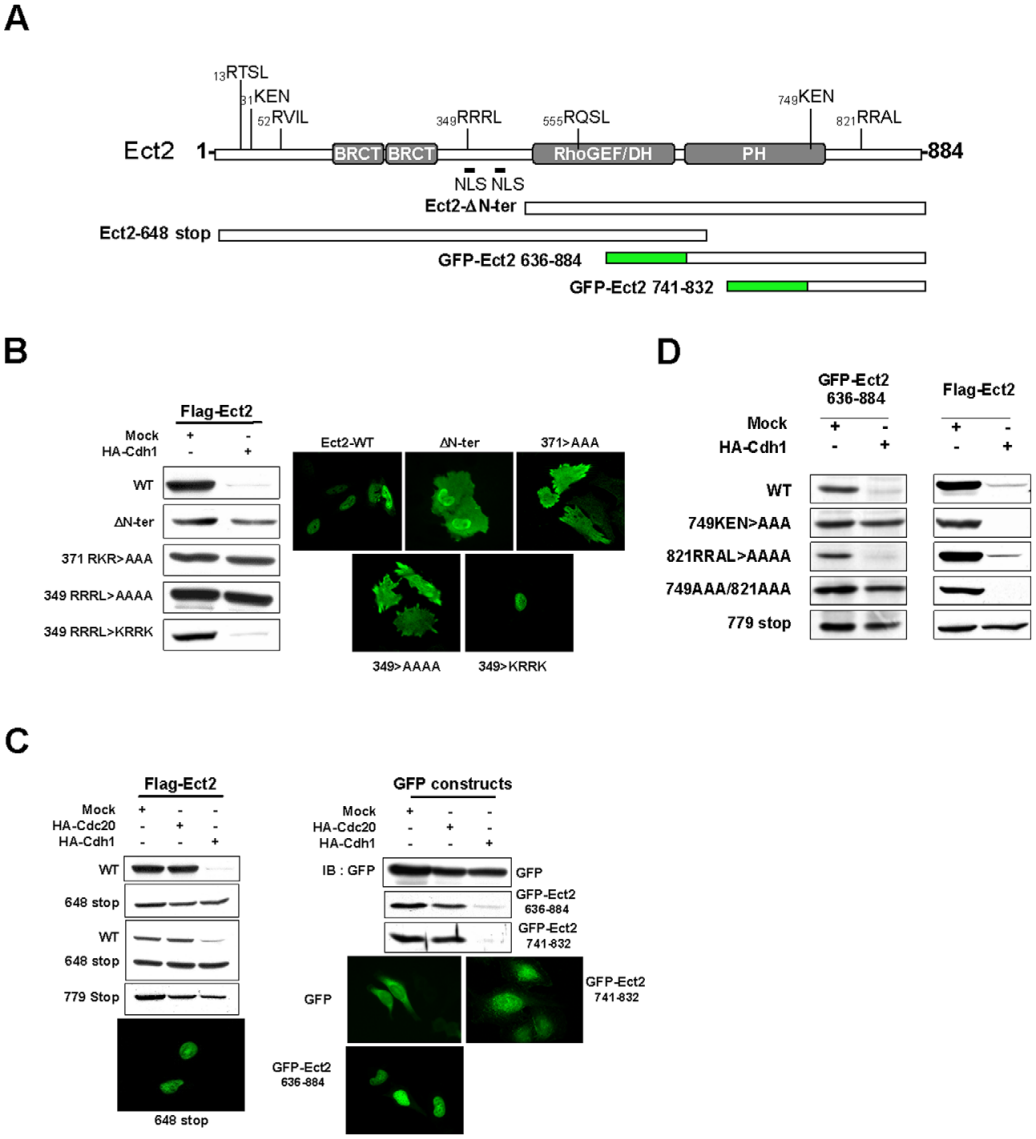
Subcellular localization and degradation motifs are involved in Ect2 degradation. (**A**) Scheme of Ect2 subdomains, degrons and constructs. (**B**) Degradation of Ect2 deleted of its N-terminus or mutated within the NLS in the presence of exogenous Cdh1 in HEK 293 cells as in Figure 2B. Subcellular localization of mutated Ect2 in Hela cells, imaged with anti-Flag antibodies following fixation in cold methanol. (**C**) Degradation of Ect2 C-terminus deletion mutants and of GFP fusion proteins containing Ect2 C-terminus as indicated. (**D**) Degradation of Flag-Ect2 or GFP-Ect2 (636–884) bearing mutations in the C-terminal D-box or KEN-box. All western blots shown here were controlled for equivalent loading of the lanes with anti-Hsc70 antibodies: the controls are provided in supplementary figure S1.

APC-mediated ubiquitination depends on either one of two rather well-defined target sequences in the substrates, the Destruction box (D-box) and the KEN box [21], [22], which are collectively referred to as “degrons”. These degrons are most often found in combination in APC substrates [23], [24], [25], [26]. Ect2 contains multiple putative KEN and D-boxes of the type RxxL (Figure 3A). We then focussed on analyzing the domain/sequence requirements for APC^Cdh1^-mediated degradation of Ect2 by generating deleted or site mutated versions of Ect2 (Figure 3). A first mutant which we found to be protected from degradation was a version of Ect2 lacking the first 375 amino acids at the N-terminus (ΔN-ter, AA 376–884). This suggested that the KEN and D-boxes at positions 13, 31, 52 and 349 might play a role in APC^Cdh1^ recognition. Ect2 is mainly localized to the nucleus of interphasic cells, due to two previously identified nuclear localization signals (NLS) starting at positions 347 and 370 [27]. In contrast, ΔN-ter Ect2 was also detected in the cytoplasm, raising the hypothesis that subcellular localization might be important for Ect2 degradation. This was assessed directly by mutating these NLS in the full length protein. Indeed, mutating the 371-RKR basic sequence to AAA resulted in delocalization of Ect2 from the nucleus and a complete stabilization of the protein (Figure 3B and supplementary Figure S2). Similarly, mutating the positively charged residues RRR(L) at position 349 to AAA(A) delocalized Ect2 and prevented its degradation by APC^Cdh1^. However, this sequence might also constitute a bona fide RxxL D-box. We therefore generated another mutant that retains a stretch of positive charges in the sequence (349RRRL to KRRK) while ablating the D-box. This mutant localized normally to the nucleus and was degraded to the same extend as wild type Ect2, indicating that this 349RRRL sequence does not represent a critical D-box but rather participates in the NLS. Taken together the results obtained with this first series of mutants demonstrated that a proper nuclear localization of Ect2 is required for its degradation by the APC.

### Characterization of degradation signals of Ect2

Another mutant Ect2 protein which we found to be resistant to Cdh1-induced degradation as compared to the wild type protein was one with a stop codon introduced at position 648 (Figure 3C, top two panels). To exclude possible artefacts, both the full length and the 648-stop constructs were then transfected together in the same cells, leading to co-expression of both proteins which can be distinguished by their different apparent molecular weight. These experiments confirmed that in the presence of exogenous Cdh1 the 648-truncated protein remained undegraded while levels of full length Ect2 were greatly reduced (Figure 3C, third panel). Importantly, this truncated version of Ect2 retained its normal nuclear localization due to the presence of both NLS in the N-terminus. Because the stop codon introduced at position 648 truncates the pleckstrin homology (PH) domain (AA636–763) we wondered whether the PH domain was important for Ect2 degradation. This seems not to be the case as an additional mutant truncated at position 779 was also resistant to degradation. Furthermore, mutating a conserved tryptophan residue at position 752, that is critical for the function of PH domains, did not affect degradation of Ect2 (supplementary Figure S3).

**Figure 4:**
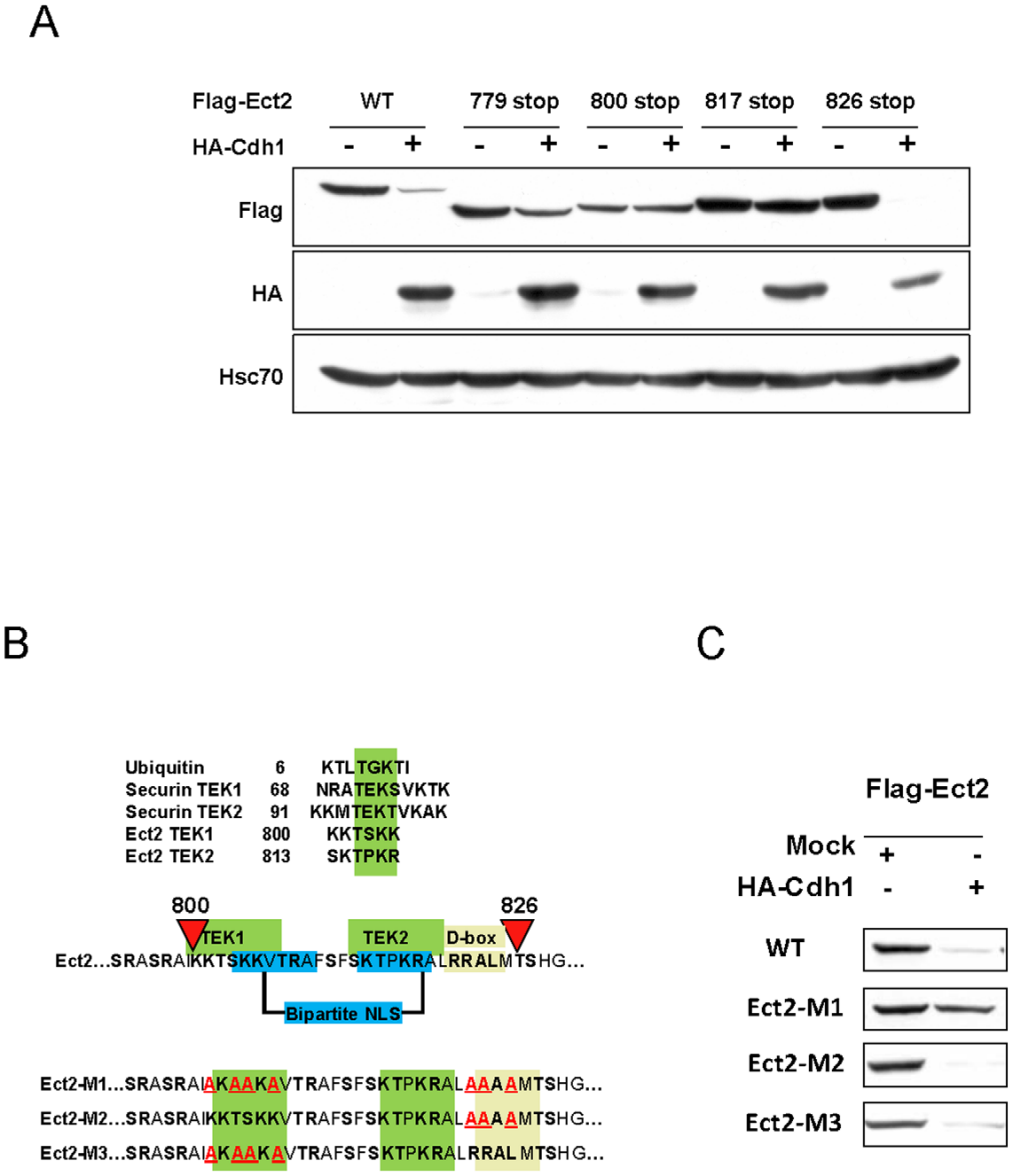
Identification of a critical, multi element signal for Ect2 degradation. (**A**) Degradation of C-terminal truncation mutants of Ect2. Subcellular localization of these mutants is shown in supplementary Figure S4. (**B**) Characterization of a bipartite NLS and of TEK-like boxes in Ect2 and (**C**) The Ect2-M1 construct with combined mutations in TEK1- and D-boxes is resistant to Cdh1-induced degradation.

To investigate the role of the C-terminus end of Ect2 in its degradation, we first fused Ect2 residues 636–884 to GFP. In contrast to wild type GFP, this GFP-Ect2(636–884) protein was localized to the nucleus in Hela cells and became susceptible to Cdh1-induced degradation (Figure 3C). Thus the C-terminus of Ect2 contains both nuclear localization signals and sequences that are able to target GFP for Cdh1-initiated degradation. The C-terminal sequence of Ect2 indeed contains a putative KEN box at position 749 and a D-box at position 821. We introduced mutations at these residues in the GFP-Ect2 (636–884) and in the full length protein as shown in Figure 3D. Mutation of 749KEN to AAA significantly reduced degradation of the GFP-Ect2 (636–884), yet mutation of 749KEN alone appeared insufficient in the context of the full length protein suggesting that, if it is involved in Cdh1 recognition, it is not by itself a critical element. On the other hand, mutations of the 821RRAL D-box had no effect whether mutated alone or in combination with the KEN-box mutation. Analysis of additional C-terminal deletion mutants clearly indicated that residues critical for Ect2 degradation were present between AA 817 and AA 826 (Figure 4A). By combining visual examination with computer searching for motifs (Motif Scan at http://www.expasy.org), the sequence between AA 800 and AA 826 appears to contain many interesting features: 1) stretches of positively charged residues predicted to constitute a bipartite NLS [28], 2) The 821RRAL D-box discussed above 3) TEK-like boxes, that have been described by Jin and colleagues as novel motifs that facilitate ubiquitin chain nucleation in APC substrates [18], 4) serine and threonine residues that are either predicted to be, or have actually been found phosphorylated in mitosis [29].

Since mutation of the 821D-box alone had no detectable effect on Ect2 degradation, we wondered whether adjacent residues might be involved in targeting the APC. Indeed, this sequence contains two regions that resemble TEK-boxes (Figure 4B). TEK-boxes are present in ubiquitin, but also in APC substrates such as securin where they are in a close proximity to a D-box [18]. We generated a large series of mutants with alanine substitution at one or several positions in this region. Only one of these mutants (Ect2-M1), that combined mutations in TEK1 with mutations of the D-box, was resistant to Cdh1-induced degradation (Figure 4C). In contrast Ect2 proteins that only contain mutations in the D-box (Ect2-M2) or in the TEK1 box (Ect2-M3) were normally degraded in our assay. Thus, the sequence 800–826 in Ect2 represents a multi element destruction signal containing classical degrons, TEK-like boxes and a bi-partite NLS. Of interest, Ect2 proteins mutated in the 800–826 region were mostly localized in the nucleus of interphasic cells, probably by virtue of the two N-terminal NLS (supplementary Figure S4). However, the third bi-partite NLS identified at AA residues 804–809/814–818 was found to be fully functional as it was indeed able to target GFP to the nucleus and shorter GFP-Ect2 constructs not containing this region were delocalized to the cytoplasm (eg GFP-Ect2 636–817, supplementary Figure S5). Whether the function of this NLS may be either cryptic or regulated by phosphorylation during mitosis remains to be investigated. In addition, the possibility that some of these lysine residues may act as substrate lysine for ubiquitin attachment cannot be excluded.

### Non degradable Ect2 transforms NIH3T3 cells

**Figure 5:**
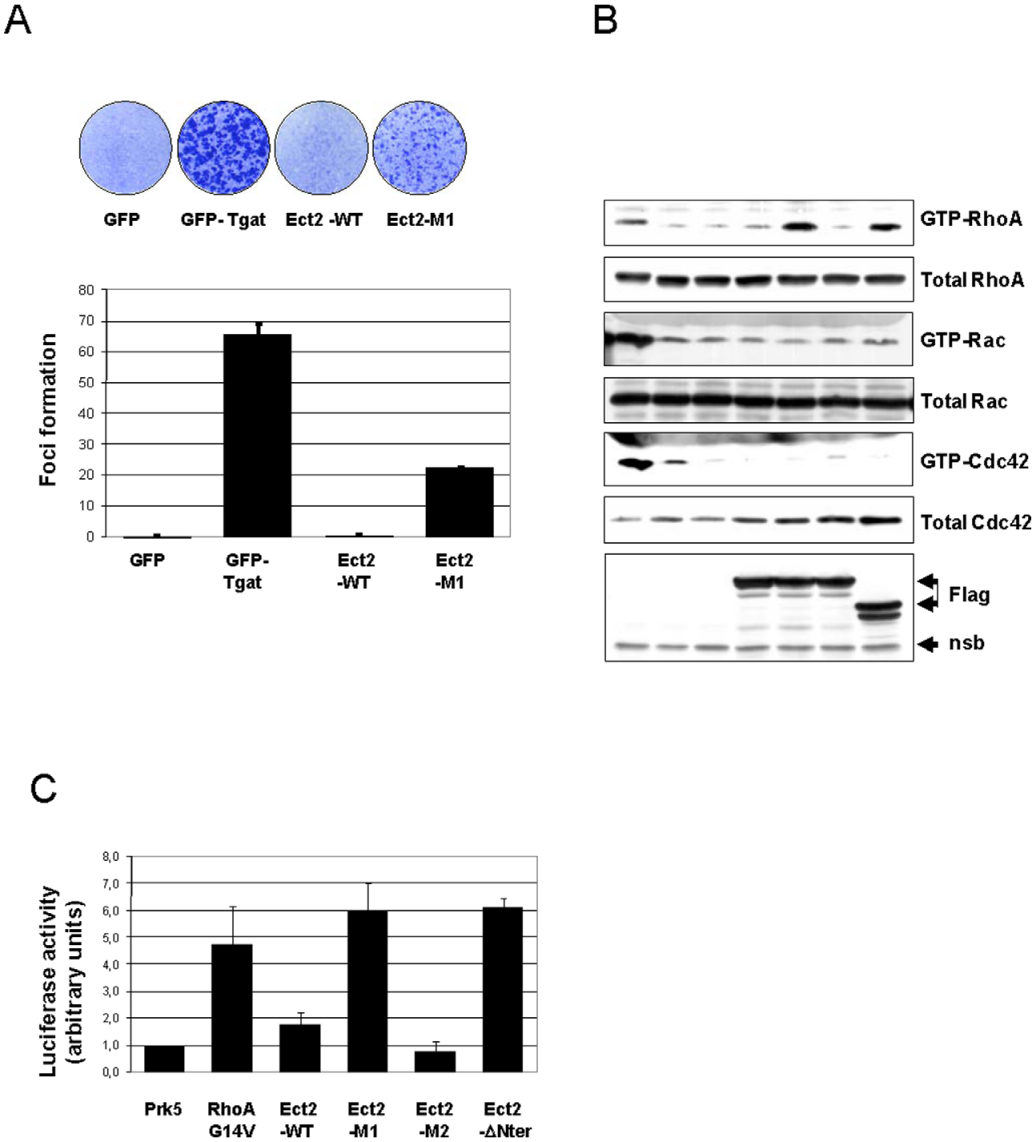
Transforming activity of Ect-2 proteins. (**A**) Retroviruses directing expression of wild type Ect2 or mutated Ect2 as indicated were used to transform NIH3T3 cells and foci were analyzed after 20 days in culture. Viruses directing expression of GFP or of Tgat, a spliced version of the RhoGEF TRIO, were used as negative and positive controls respectively. Foci were scored using the Metamorph software: the results shown are means from two independent experiments each performed in triplicate. Error bars indicate standard deviation of the mean. (**B**) Rho GTPase activation by Ect2 mutants: Hela cells were transfected with the indicated constructs. After 24 h of culture, cell lysates were prepared as described in materials and methods, and processed for determining the level of activated GTPases, using GST-Rhotekin RBD for Rho, or GST-PAK CRIB for Rac and Cdc42. Activated GTPases pulled down in this assay were revealed with the indicated antibodies. (**C**) HEK 293 cells were cotransfected with SRE-luciferase (firefly) reporter and pSR*α*-*Renilla* standardization plasmids together with the indicated expression vectors. Results were normalized by dividing firefly signals by the renilla signals, and activity obtained with empty prk5 expression plasmid was set to one. The results shown are means from three independent experiments. Error bars indicate standard deviation of the mean.

Ect2 was initially described as an oncogene [30] and N-terminal truncated versions of Ect2 are able to transform NIH3T3 cells, a property that has been associated with their abnormal cytoplasmic localization and ability to activate RhoA or Rac1 [27], [31]. We show here that N- terminal truncated Ect2 is not only mislocalized but also resistant to APC^Cdh1^-mediated degradation. We hypothesized that besides a cytoplasmic localization of Ect2 deleted of its major N-terminal NLS, the resulting stabilization of the protein may also contribute to its transforming ability. If this were the case, then other non degradable mutants of Ect2 should also be oncogenic. Indeed as shown in Figure 5A the Ect2-M1 construct bearing mutations that prevent its degradation was able to induce transformation foci in NIH3T3 cells. The transforming activity of Ect2 is thought to be mediated via activation of Rho GTPases and we indeed found an increase in Rho-GTP in Hela cells transiently transfected with the Ect2-M1 construct (Figure 5B). Furthermore, Ect2-M1 was able to drive luciferase production from a cfos-SRE reporter plasmid to the same levels as an activated mutant of RhoA(G14V) or the N-terminal truncated Ect2 (Figure 5C).

## Discussion

In mitosis, Ect2 and MgcRacGAP are essential regulators of RhoA which then acts through several effector kinases such as ROCK, citron or PRK2 to ensure contractile ring formation and cleavage furrow ingression [32], [33], [34]. Together with our previous work [15] the results presented in this study characterize Ect2 as a bona fide cell cycle-regulated protein: its expression is induced in S-phase and it then undergoes proteasomal-dependent degradation shortly after mitosis is completed. Furthermore, we identify Ect2 as a new substrate of the mitotic ubiquitin ligase APC^Cdh1^. Of interest, it has recently been reported that degradation of p190RhoGAP was required for exit of mitosis [35]. Although the authors did not characterize the ubiquitin ligase involved in this process, APC^Cdh1^ stands as a likely candidate since a separate group demonstrated targeting of p190RhoGAP by APC^Cdh1^ in postmitotic or quiescent cells [36]. Thus, notwithstanding the critical importance of protein phosphorylation or subcellular localization, regulation of RhoGEFs and RhoGAPs at the level of protein expression by APC^Cdh1^-mediated degradation may represent a central event in controlling Rho activity during mitosis.

The currently accepted models for substrate recognition by APC/C describe the role of KEN and/or D-boxes in the substrate sequence as binding sites for Cdc20 or Cdh1. The Ect2 protein sequence contains 2 putative KEN boxes and 5 D-boxes of the type RxxL. However, despite our efforts we have not been able to identify any of these putative target boxes that, alone, would appear either sufficient or critical to control Ect2 degradation. It is possible that two or more of these individual degrons are required for binding to APC and that we missed the right combination in our attempts. One additional level of complexity is that at least two of these D-boxes are embedded within nuclear localisation signals, and we have found that a proper nuclear localisation of Ect2 was required for its degradation. An alternative explanation suggested by our data is that the D-box located at position 821 of the protein sequence, cooperates with the first of the two putative TEK-like boxes in this region of Ect2. As described by Jin et al. [18], TEK boxes identified in APC substrates, such as securin, promote recognition of an adjacent D-Box and facilitate ubiquitin chain nucleation. As far as we understand it today, this mode of interaction between APC and its substrates would then favour elongation of K11-linked poly-ubiquitin chains. Indeed, by co-expressing vectors for ubiquitin bearing mutations on specific lysine residues, we found that a majority of ubiquitin chains on Ect2 would be K11-linked.

Ubiquitination of many proteins that are involved in mitosis or cytokinesis is often regulated by phosphorylation. In that respect, Ect2 has been found to be hyper-phosphorylated in mitosis notably by CDK1, and our data (Fig. 1B) suggest that the phosphorylated form of Ect2 might be degraded more readily than the non-phosphorylated form. However, Ect2 contains at least 18 predicted sites for phosphorylation by serine/threonine kinases. We have attempted to target several of these sites, including those that have been described (eg T342) to play a role in the biology of Ect2, by replacing critical threonine residues either by alanine or aspartic acid residues to generate phosphomimetic Ect2 mutant. However, these experiments have so far not allowed us to identify a control of Ect2 degradation by phosphorylation at these sites, and additional work is required to clarify this issue.

It is unclear at this point whether, in normal cells, Ect2 is involved in regulating RhoA in response to extracellular cues or whether it is solely recruited, through phosphorylation or activating interactions, during mitosis. If this was indeed the case then regulating its protein levels would prevent improper or untimely activation of the Rho pathways. It is of interest that Ect2 was recently reported to be overexpressed in various human cancers where it correlates with tumor aggressiveness and poor outcome prognosis [37], [38], [39], [40]. The mechanisms whereby Ect2 overexpression is achieved in tumor cells are likely to be multiple, including gene amplification [39] or transcriptional up-regulation, for instance through aberrant activation of the pRb/E2F pathway as indicated by our unpublished observations and other reports [41]. In addition, the work reported here suggests that impaired Ect2 degradation might also be responsible for increased expression. Indeed, it has been found that a number of APC/C substrates are frequently overexpressed in malignant tumors [42] and, given its role in preventing genomic instability, Cdh1 has been proposed as a tumour suppressor [43]. Ect2, as an oncogene, could turn out an important Cdh1 target in this context. In addition, it has recently been reported that Ect2 is overexpressed and mislocalized to the cytoplasm in human non small cell lung cancer, as a result of phosphorylation at residue T328 by PKCι [39], [44], Since we showed here that nuclear localization of Ect2 is required for its degradation at mitosis exit, it is possible that T328-phosphorylated Ect2 may escape degradation. While this work was in progress, we also became aware that a mutation targeting the C-terminus of Ect2, namely replacing T802 with P, had been described in the breast cancer-derived cell line Hcc38 (The Sanger Institute Catalogue Of Somatic Mutations In Cancer web site, http://www.sanger.ac.uk/cosmic [45]). In preliminary experiments (not shown) we found that replacing T802 by an alanine residue had no effect on its sensitivity to Cdh1-induced degradation, whereas replacing it with proline indeed rendered Ect2 resistant to Cdh1-dependent degradation in our HEK 293 assay. This is likely the consequence of the structural constraints imposed by proline versus alanine that might disrupt either Ect2 interaction with the APC/Cdh1 complex or Ect2 ubiquination by the E3 ligase, and suggests that it is worth investigating further whether Ect2 stabilizing mutations are to be found in human cancers.

## Materials and Methods

### Cell lines and culture conditions

The human IL2-dependent Kit 225 T-cell line [46] was maintained in RPMI 1640 culture medium containing 2 mM L-glutamine, antibiotics, 2% sodium pyruvate, 10% fetal calf serum and 0.5 nM recombinant human IL-2 (Proleukin, Chiron corp., The Netherlands). Kit 225 cells can be synchronized in G1 by culture in the absence of IL-2 for 48 hrs [15]. The HEK 293 and Hela cell lines were obtained from ATCC. NIH3T3 [47], HEK 293 and Hela cell lines were cultured in D-MEM supplemented with antibiotics and 10% fetal calf serum. Kit 225 and HEK 293 cells were synchronized in G1/S by a double thymidine block: cells were treated by 2 mM thymidine (Calbiochem) for 16 hours, then washed and released for 9 hours in fresh medium. Cells were again treated by 2 mM thymidine for an additional 16 hours, and cell cycle progression was induced by washing the cells and releasing in fresh medium. For synchronization in mitosis, cells were treated for 14–16 hrs with nocodazole (Sigma-Aldrich, 40 ng/mL) then washed and reseeded in culture medium. Cell DNA content was analyzed by flow cytometry following propidium iodide staining and analysis of the fraction of cells in G1, S or G2/M was performed using the Modfit software.

### Plasmid constructs

Human Ect2 cDNA was amplified by PCR from Kit 225 mRNAs, tagged with sequences encoding the Flag epitope and inserted in the multiple cloning site of the pRK5 expression plasmid using standard molecular biology procedures. Site directed mutagenesis was performed using the Quikchange mutagenesis kit (Stratagene, La Jolla, CA) according to the manufacturer's instructions. All constructs, whether wild type or mutated, were verified by DNA sequencing.

Plasmids directing the synthesis of ShRNA targeting Cdh1 or Cdc20 [20] were provided by R. Agami (Division of Tumor Biology, The Netherlands Cancer Institute). Expression vectors for HA-Cdh1 and HA-Cdc20 [48] were provided by ZF Chang (Institute of Biochemistry and Molecular Biology, Taipei). The ubiquitin pRBG4-6HisUb expression vector [49] was provided by E. Lemichez (Inserm U627, Nice).

Transient transfections of HEK 293 or Hela cells were performed using Jet-PEI (Ozyme) and various amounts of DNA. Unless specified otherwise, cells were collected 24 h after transfection.

### Ubiquitination experiments

HEK 293 cells were transfected with 5 µg (for ∼10^6^ cells) of the 6HisMyc-Ubiquitin and Flag-Ect2 expression vectors, and collected for lysis after 24 hours. When indicated cells were pre-treated with MG132 (20 µM) for 6 hours before lysis at room temperature in urea buffer (20 mM Tris-HCl pH 7,5, 200 mM NaCl, 10 mM Imidazol, 0,1% TritonX-100, 8 M Urea) as described in [49]. Lysates were cleared by centrifugation and incubated with Cobalt-loaded beads (Clonetech) for 1h30 at room temperature. Bound proteins were separated by SDS-PAGE and analyzed by western blotting.

### Cdh1-mediated degradation

HEK 293 cells were transfected with 1 µg (for ∼2.5×10^5^ cells) of the Flag-Ect2 vectors and 2 µg of either pcDNA3-HA-Cdh1, pcDNA3-HA-Cdc20, or empty pcDNA3. Where indicated, 20 µM MG132 was added for the last 16 h in culture. Cells were then collected 24 h after transfection, lysed and analyzed by western blotting.

### Luciferase assay

Luciferase assays using a firefly luciferase reporter plasmid containing one copy of the SRE of human c-*fos* promoter (SRE Luc), in front of a minimal c-*fos* promoter (−90 to +42 bp with reference to the transcription start site) and pSRα-*Renilla* reporter for standardization were performed as previously described [50]. Briefly, HEK 293 cells were cotransfected with reporter plasmids and expression vectors for the various Ect2 mutants or an activated RhoA(G14V) construct using Jet PEI. The total amounts of transfected DNA were kept constant by addition of empty control vector. After 24 hours in culture, cells were lyzed in buffer and luciferase activity was measured on a MicroLumat Plus LB 96 V luminometer (Berthold Technologies, Pforzheim, Germany).

### Focus formation assay

To create stable NIH 3T3 cell lines, GFP, GFP-Tgat, Flag-tagged Ect2-wt and Ect2-M1 were cloned into the puromycin-resistant retroviral vector pBabePuro as previously described [47]. The indicated retroviral constructs were transfected into BOSC packaging cells, using the JetPEI reagent. Forty-eight hours after transfection, virus-containing supernatants were collected and used to infect NIH 3T3 cells. Infected cells were selected with 6 µg/ml puromycin and stable transfectants were pooled after selection. Flag-Ect2 protein expression levels were monitored by western blot analysis using a monoclonal anti-Flag antibody or a polyclonal anti-Ect2 antibody. Focus formation assays were performed using stable NIH 3T3 cell lines as indicated, seeded at 5×10^4^ cells in 6-well plates and maintained for 14 to 24 days in 10% fetal bovine serum. Medium was renewed every 2 days. After staining with crystal violet (1%), plates were photographed and foci were scored using the Metamorph software. All experiments were done in triplicate.

## Supporting Information

Figure S1 **Loading controls for blots shown in** **Fig. 3** **and** **Fig. 4** **.** The right handside of each panel represents reblots with anti-Hsc70 antibodies.
(TIFF)

Figure S2 **Brigth field images for fluorescence data shown in** **Fig. 3** **.**
(TIFF)

Figure S3 **The PH domain is not involved in Ect2 degradation. (A)** Scheme of C-terminus deletion mutants or KEN-box mutated Ect2 constructs. (**B**) Degradation of the above Ect2 mutants in HEK 293 cells in the presence of exogenous Cdh1. Blot Flag.
(TIFF)

Figure S4 **Nuclear localization of Ect2 proteins mutated in the AA 800–826 region.** Hela cells were transfected with the indicated constructs. After 24 h, cells were fixed in cold methanol, then stained with anti-Flag antibodies and Alexa 488-conjugated secondary anti-mouse antibodies. Images were acquired on an AxioImager Zeiss fluorescence microscope.
(TIFF)

Figure S5 **Subcellular localization of C-terminus deleted Flag-Ect2 proteins or GFP fusion constructs in Hela cells.** Hela cells were transfected with the indicated constructs, fixed in cold methanol and either observed directly for GFP or processed as in Figure S4.
(TIFF)
