# Supplementary figures and images for: APCcdh1 Mediates Degradation of the Oncogenic Rho-GEF Ect2 after Mitosis
Source: PLoS One. 2011 Aug 19;6(8):e23676. doi: 10.1371/journal.pone.0023676 (PMC3158779; doi:10.1371/journal.pone.0023676)

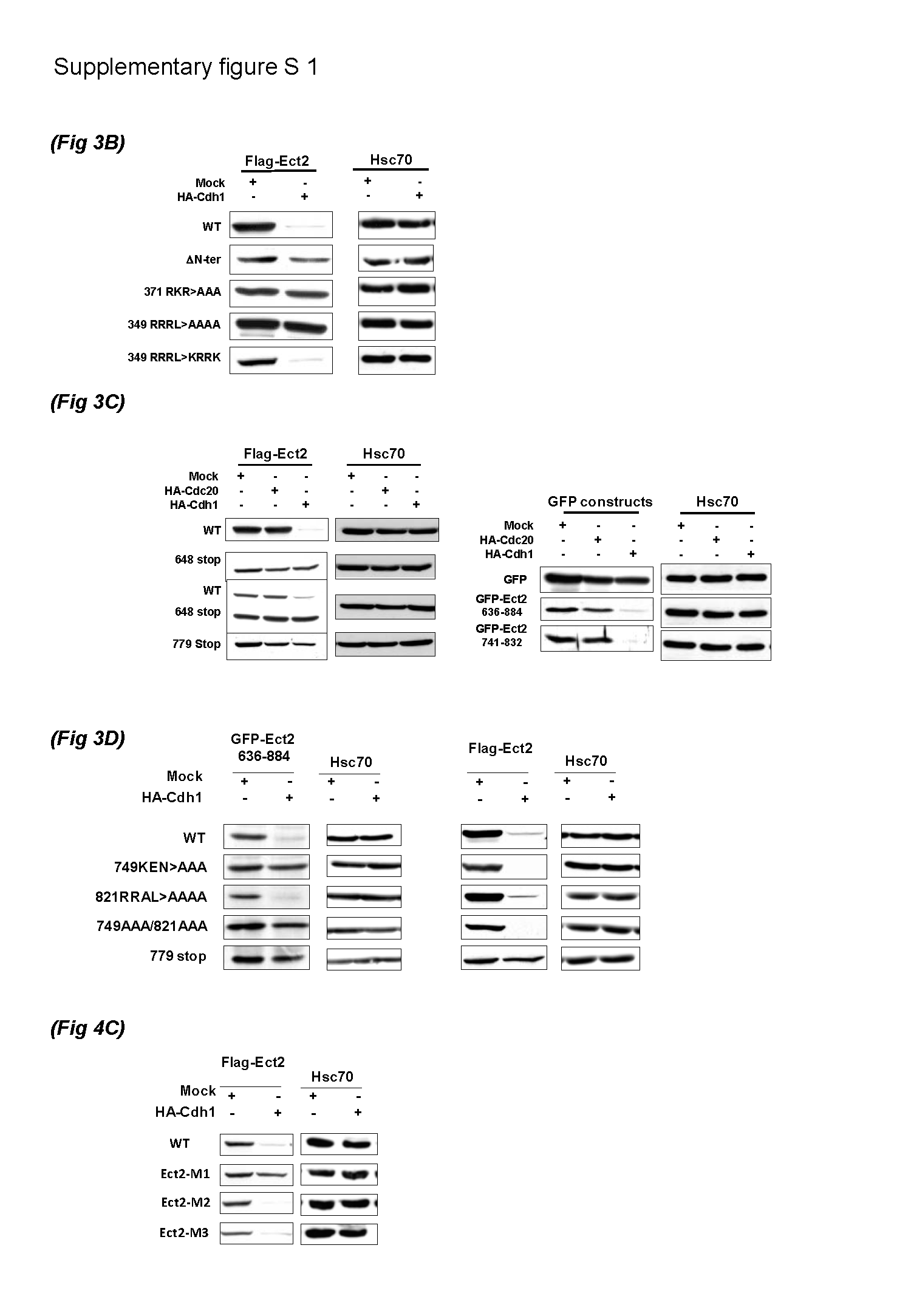

Supplement: Figure S1 — Loading controls for blots shown in Fig. 3 and Fig. 4 . The right handside of each panel represents reblots with anti-Hsc70 antibodies. (TIFF) [file pone.0023676.s001.tiff]

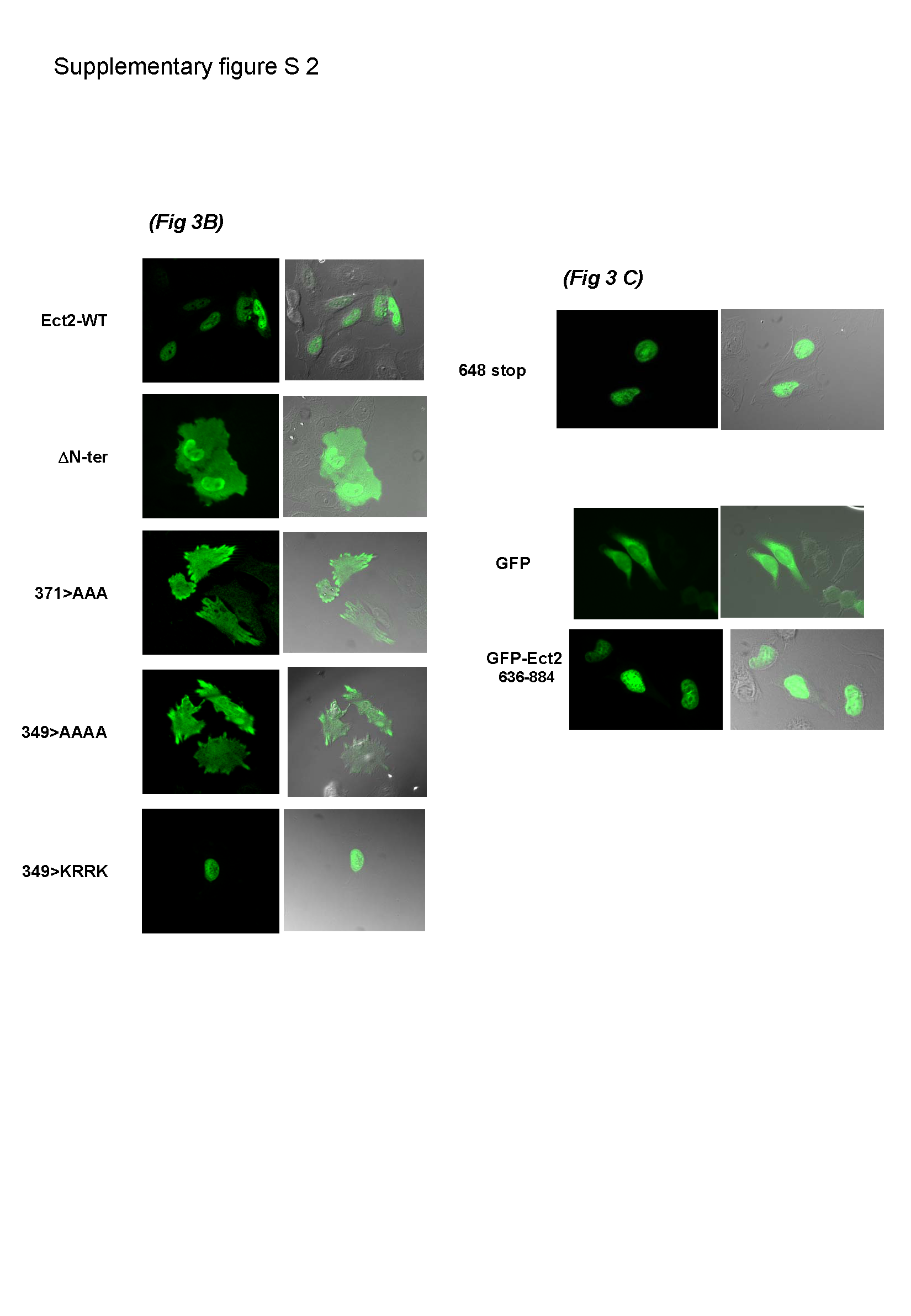

Supplement: Figure S2 — Brigth field images for fluorescence data shown in Fig. 3 . (TIFF) [file pone.0023676.s002.tiff]

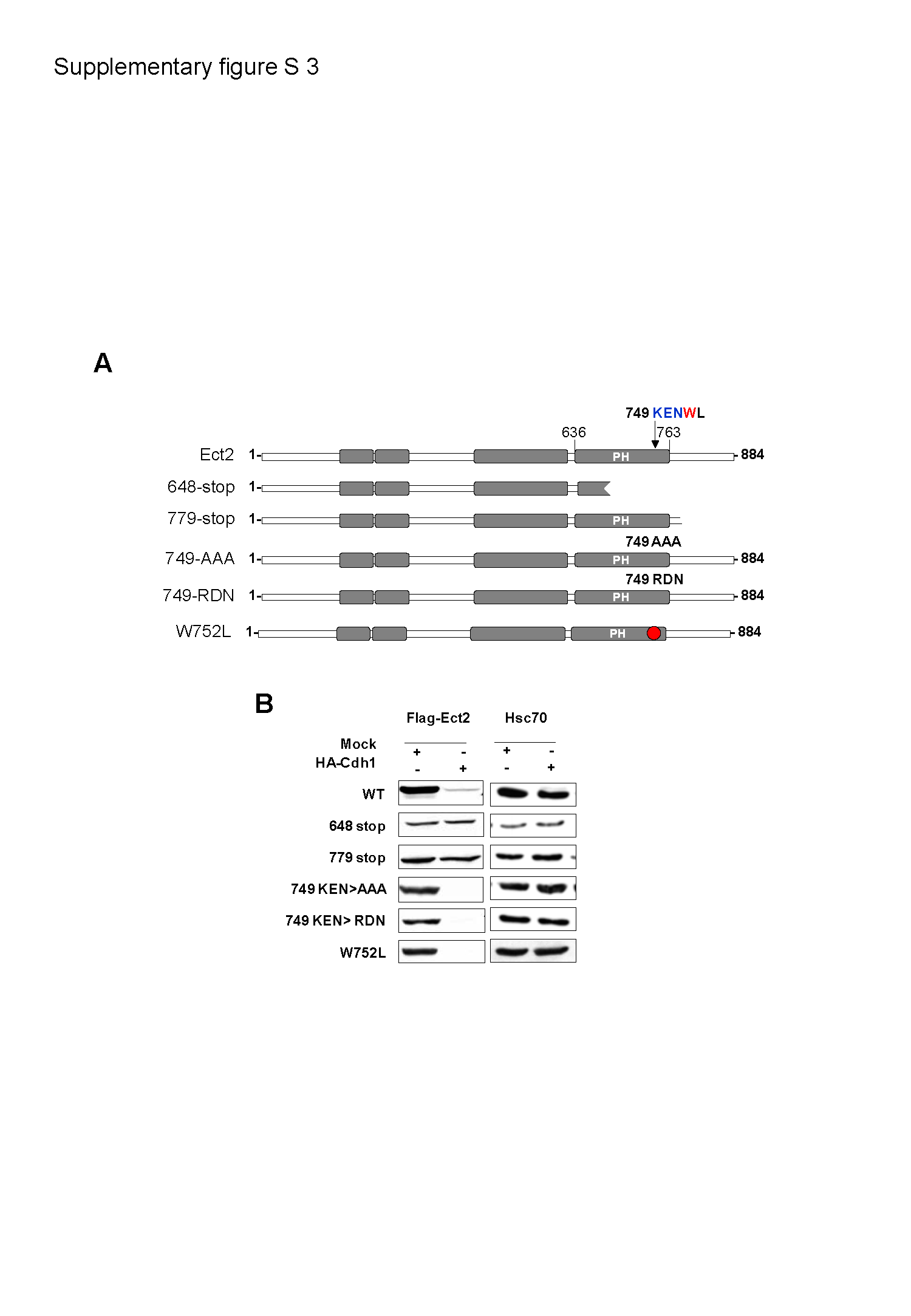

Supplement: Figure S3 — The PH domain is not involved in Ect2 degradation. (A) Scheme of C-terminus deletion mutants or KEN-box mutated Ect2 constructs. (B) Degradation of the above Ect2 mutants in HEK 293 cells in the presence of exogenous Cdh1. Blot Flag. (TIFF) [file pone.0023676.s003.tiff]

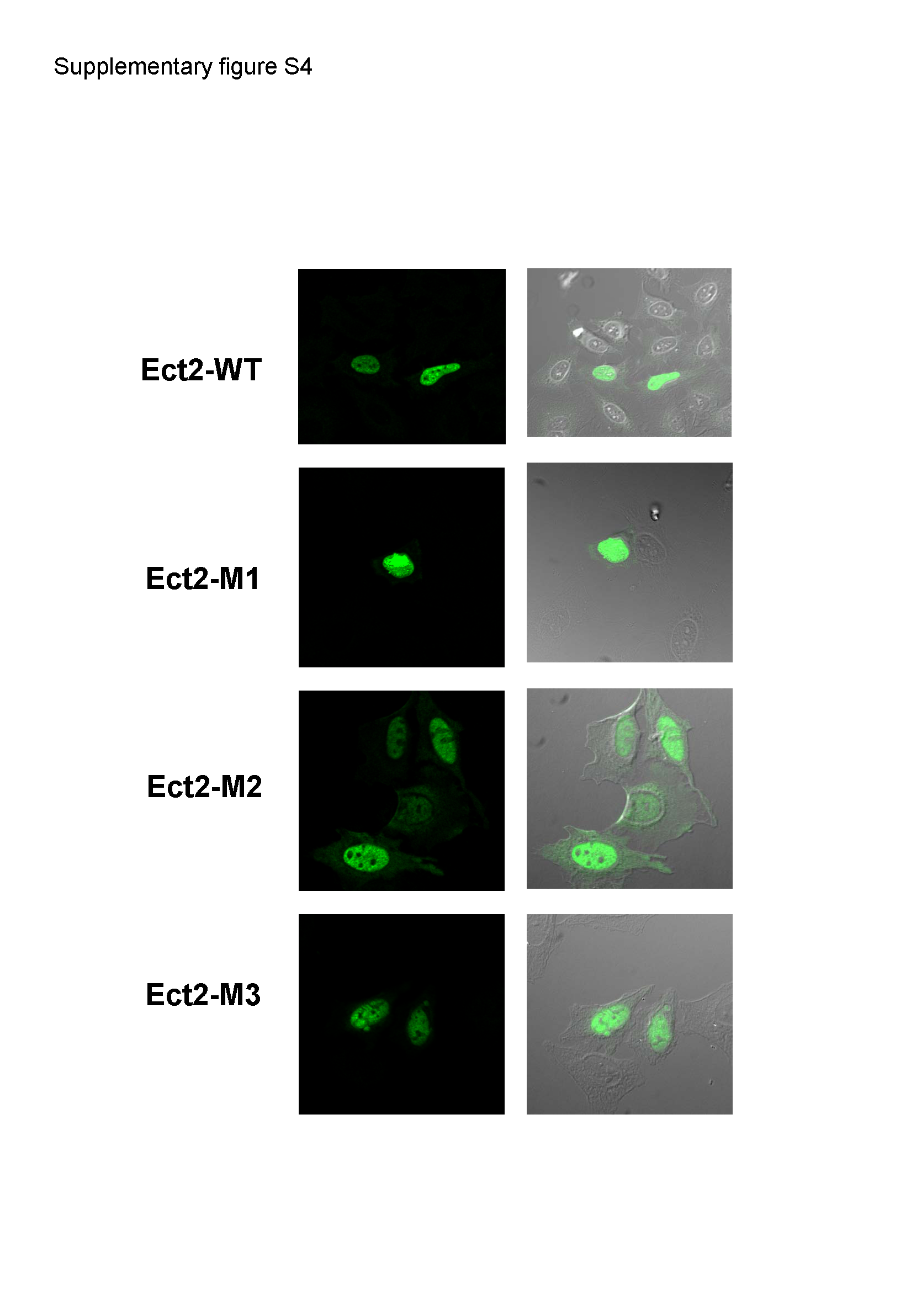

Supplement: Figure S4 — Nuclear localization of Ect2 proteins mutated in the AA 800–826 region. Hela cells were transfected with the indicated constructs. After 24 h, cells were fixed in cold methanol, then stained with anti-Flag antibodies and Alexa 488-conjugated secondary anti-mouse antibodies. Images were acquired on an AxioImager Zeiss fluorescence microscope. (TIFF) [file pone.0023676.s004.tiff]

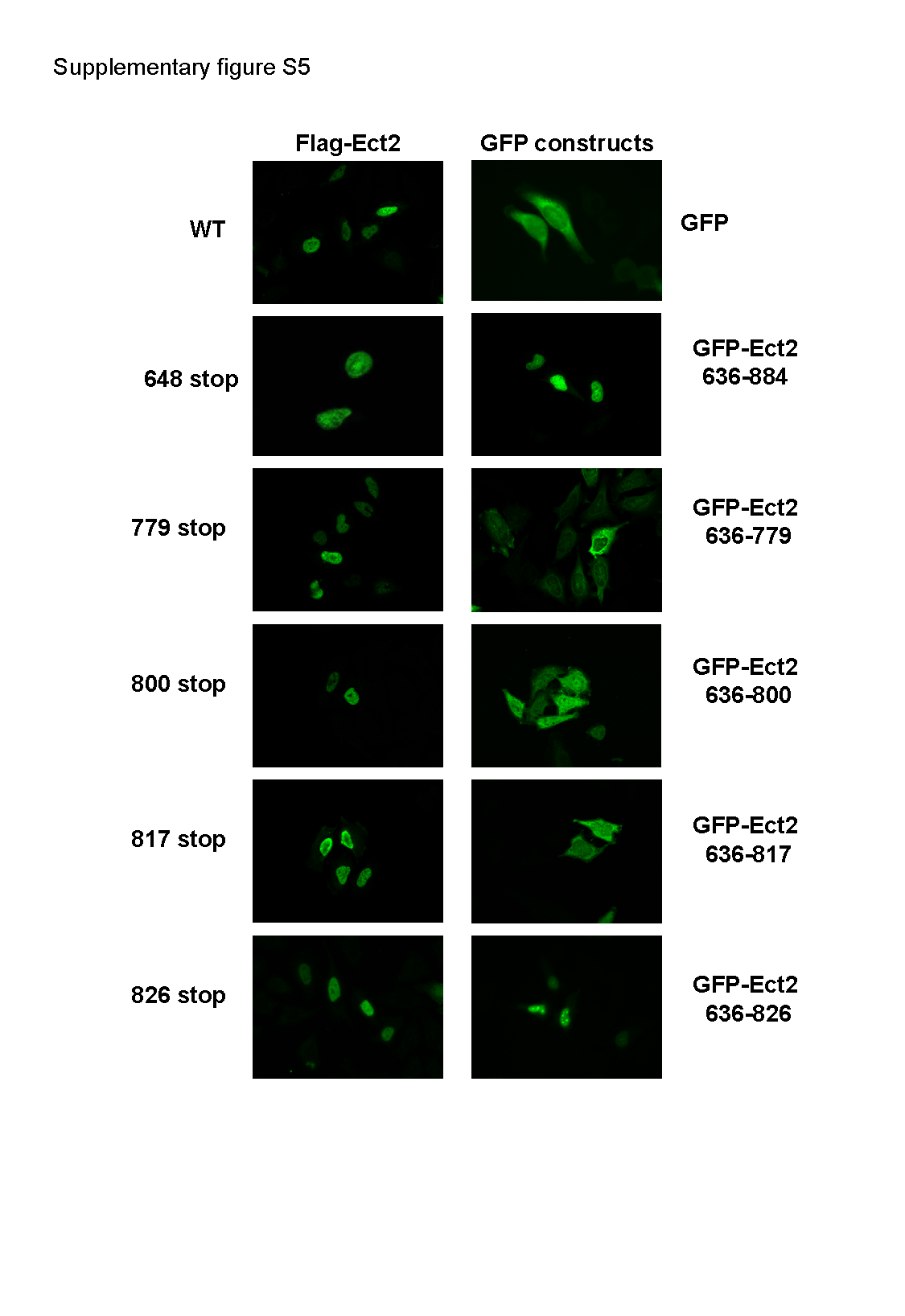

Supplement: Figure S5 — Subcellular localization of C-terminus deleted Flag-Ect2 proteins or GFP fusion constructs in Hela cells. Hela cells were transfected with the indicated constructs, fixed in cold methanol and either observed directly for GFP or processed as in Figure S4. (TIFF) [file pone.0023676.s005.tiff]
